# New Highly Sensitive and Specific Raman Probe for Live Cell Imaging of Mitochondrial Function

**DOI:** 10.1021/acssensors.3c02576

**Published:** 2024-02-09

**Authors:** Anna Pieczara, Ruben Arturo Arellano Reyes, Tia E. Keyes, Patrycja Dawiec, Malgorzata Baranska

**Affiliations:** † Jagiellonian Centre for Experimental Therapeutics (JCET), Jagiellonian University, 14 Bobrzynskiego Str., 30-348 Krakow, Poland; ‡ Jagiellonian University in Kraków, Doctoral School of Exact and Natural Sciences, 11 Lojasiewicza Street, 30-348 Krakow, Poland; § Faculty of Chemistry, Jagiellonian University, 2 Gronostajowa Str., 30-387 Krakow, Poland; ∥ School of Chemical Sciences, Dublin City University, 592, 628 Collins Ave Ext, Whitehall Dublin 9, D09 E432 Dublin, Ireland

**Keywords:** mitochondrial membrane, Raman probe, RAR-BR, CCCP, spontaneous Raman microscopy, fluorescence microscopy

## Abstract

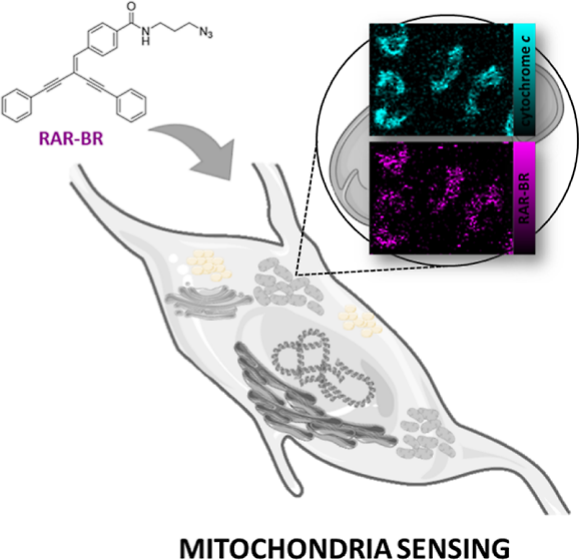

For Raman hyperspectral detection and imaging in live cells, it is very desirable to create novel probes with strong and unique Raman vibrations in the biological silent region (1800–2800 cm^–1^). The use of molecular probes in Raman imaging is a relatively new technique in subcellular research; however, it is developing very rapidly. Compared with the label-free method, it allows for a more sensitive and selective visualization of organelles within a single cell. Biological systems are incredibly complex and heterogeneous. Directly visualizing biological structures and activities at the cellular and subcellular levels remains by far one of the most intuitive and powerful ways to study biological problems. Each organelle plays a specific and essential role in cellular processes, but importantly for cells to survive, mitochondrial function must be reliable. Motivated by earlier attempts and successes of biorthogonal chemical imaging, we develop a tool supporting Raman imaging of cells to track biochemical changes associated with mitochondrial function at the cellular level in an *in vitro* model. In this work, we present a newly synthesized highly sensitive RAR-BR Raman probe for the selective imaging of mitochondria in live endothelial cells.

Mitochondria are small cytoplasmatic organelles, 0.5 to 1.0 μm in diameter, crucial in signaling cell death and survival.^1^ They are responsible for adenosine triphosphate (ATP) synthesis,^2–4^ the creation of reactive oxygen species (ROS) and free radicals (FR).^5,6^ Mitochondria also play key roles in the production of cellular building blocks, amino acids, and fatty acids.^7^ Additionally, mitochondria take part in cell signaling,^8^ cell cycle control,^7^ and metabolism regulation.^8^ The mitochondria have a dual membrane enclosing its structure, a porous outer membrane, and an inner membrane that is highly impermeable to most ions and molecules. Molecules and ion entrance is permitted only in the presence of specific and selective membrane transporters. The inner membrane houses the respiratory chain complexes. Soluble enzyme components are present in the matrix space bounded by the inner membrane and are necessary for some mitochondrial functions.^1,9–12^ There are morphological and functional differences between mitochondria of various organs, e.g., mitochondria in endothelial cells (ECs) occupy only 2 to 6% of the cytoplasmic volume,^13^ while in cardiac myocytes, they constitute as much as 32%.^14^ Mitochondria are dynamic organelles, they can move in the direction of an area where more energy is needed as a result of cytoplasmic movements or by binding to cytoskeleton components.^15^

Directly visualizing the structure and activity of mitochondria remains a challenge. The method of choice is fluorescence microscopy (FM), but the size of the mitochondria falls below the limit of diffraction. Electron microscopy (EM) overcomes this limitation but is constrained when it comes to imaging of live cells.^12^ The sample should be dry and stable for the measurements.^16,17^ EM allows for precise determination of the architecture of mitochondrial membranes, and it is also possible to show to some extent, the distribution of proteins but only in fixed samples.^18^ Recently several super-resolution and nanoscopic microscopies have surfaced, to investigate the unknown mechanism of mitochondria activity with a subdiffraction resolution.^12,19,20^ For example, stimulated emission depletion nanoscopy is employed to study oxidative phosphorylation (OXPHOS),^21^ mitochondria proteins,^22^ and mitochondria interaction with endoplasmic reticulum;^23^ single-molecule localization microscopy is used to track subunits of OXPHOS;^24^ and three-dimensional structured illumination microscopy allows imaging of the activity of the mitochondria.^25^ Resolving mitochondria in imaging of live cells can be accomplished by immunostaining using highly specific probes to reduce background. Most of the fluorophores used to label mitochondria to date have been cationic, their permeation across the mitochondrial membrane is facilitated by the negative membrane potential of this organelle.^26^ Commercially available dyes used to label the mitochondria include, e.g., Rhodamine 123, Rosamines, or MitoTracker probes. The limitation of the FM in studies of live cells is 1/the photobleaching of fluorescent probes, 2/their toxicity and phototoxicity, and 3/the limited spatial resolution of the method.^27^ Furthermore, fluorescent dyes that accumulate in the mitochondria due to the Nernstian effect can be easily washed out of cells once the mitochondria membrane potential is lost. Also, there are commercially available dyes that form covalent bonds with proteins present on the mitochondrial membrane, but they are also not ideal due to the fact that they may interfere with the respiratory activity of mitochondria on prolonged incubation time and at high concentration.^12^ Moreover, at high concentrations, organic fluorophores tend to aggregate and stain other organelles.^28^ Also, some examples of selective and specific probes for imaging of other processes taking place in the mitochondria, for example, changes in endogenous H_2_S in living cells^29^ or oxidative stress,^30^ are reported. In addition to fluorescence imaging, luminescent probes based on iridium complexes are also used to visualize mitochondria. E.g., IraZolve-Mito can be used for the imaging of mitochondria both in live and fixed samples.^31^ Another luminescence sensor is Ir3.^28^

An attractive alternative to FM for cell imaging is Raman microscopy (RM), which in principle can be used as a label-free method.^32–34^ Raman spectra of cells are complex since they contain information about all of the molecules present in the sample, providing insights into the chemical structure and processes.^34^ Label-free imaging of mitochondria has been shown by employing confocal RM to detect the resonant Raman signal of cytochrome *c* (750 cm^–1^),^35^ an endogenous protein abundant in the mitochondrial membrane.^36^ It was possible to follow the apoptosis process of cell^37^ and identify the progress of sepsis.^38^ Monitoring the mitochondrial redox state during sepsis can provide rapid diagnostic tools other than already used blood lactate levels. By inducing apoptosis in HeLa cells, significant differences in the distribution of cytochrome *c* and change in mitochondrial membrane potential were observed. However, it is very important to note that the distribution and concentration of cytochrome *c* vary with cell type, so it is not always a reliable probe. Moreover, employing an excitation laser with a wavelength of 532 nm, cytochrome in its reduced form does not give resonance enhancement, under the same conditions as in its oxidized form.^39^ An alternative, potentially more robust approach to RM is to use so-called Raman probes (Rp) to increase the selectivity and sensitivity of Raman imaging. Labeled RM takes advantage of the silent Raman spectral region (1800–2800 cm^–1^), where no characteristic signal from biomolecules occurs.^40–42^ For this purpose, mitochondria-targeted moiety lipophilic triphenylphosphonium cation (TTP^+^) combined with bisarylbutadiyne (BADY) (MitoBADY) is most often used. The signal from MitoBADY colocalizes with cytochrome *c*(43) when it is used in low concentration and short incubation time to avoid nonspecific accumulation.

Motivated by earlier attempts and successes of biorthogonal chemical imaging, the new platform has recently been developed by combining stimulated Raman scattering (SRS) microscopy with small Rp. Such an optical imaging scheme offers a combination of high detection sensitivity and specificity, low disturbances, and dynamic analysis capability, by presenting a hybrid strategy between the traditional fluorescence microscopy and the label-free vibrational imaging approach.^44–49^ For SRS, a few more mitochondrial Rps can be found, that are based on TPP^+^ cation, like Mito-AZO,^50^ Carbow2226 Mito, Carbow2141 Mito,^49^ and MARS2237.^48^ However, due to the problems associated with the nonspecific accumulation of these Rp, they offer limited sensitivity and specificity; thus, further research is conducted to design new Rp.

In this work, we present a newly synthesized highly sensitive *N*-(3-azidopropyl)-4-(4-phenyl-2-(phenylethynyl)but-1-en-3-yn-1-yl)benzamide (RAR-BR) Rp for the selective imaging of mitochondria in live ECs. To the best of our knowledge, this is the first report of Rp based on cross-conjugated alkyne systems.

## Methods

### Cell Culture

To investigate the subcellular distribution of RAR-BR human aortic endothelial cells (HAECs) chosen as a model, the cell line was obtained from the American Type Culture Collection (ATCC, USA). HAEC were cultured and grown at 37 °C in continuously humidified air with 5% CO_2_ concentration in the supplemented EC growth EGM-2MV medium (Lonza). Approximately 24 h before the incubation HAEC were seeded directly onto CaF_2_ slides (Crystran Ltd., UK) in an amount of about 150,000 per slide for the RM measurement to give cells enough time to multiply and spread so they could attain optimal confluence. Cells were kept in a complete EGM-2MV medium and left to grow in the incubator. Prior to RM measurement, cells were treated with RAR-BR (concentration: 50 nM–5 μM, incubation time: 5–60 min), MitoBADY (concentration: 400 nM, incubation time: 15 min), and CCCP (concentration: 0.7 μM, incubation time: 15 min).

### MTT Test

Cell viability was evaluated by employing the 3-(4,5-dimethyl-2-thiazolyl)-2,5-diphenyl-2*H*-tetrazolium bromide MTT (Sigma-Aldrich). For this test, cells were seeded on a 96-well plate. The cells were incubated for 15 min without (control sample) or with RAR-BR and MitoBADY at concentrations 50, 100, and 400 nM. After sufficient incubation time, 20 μL of MTT solution was added to each well immediately to obtain 20% MTT concentration, and the cells were incubated in this way for 3 h at 37 °C. The medium was then removed, and the plate was placed at −20 °C for another 24 h. In the next step, 100 μL of isopropanol: hydrochloric acid solution was added to each well, and then, the plate was placed in a plate shaker for 30 min to react. By using a Synergy 4 plate reader (Biotek, VT, USA), the absorbance of such prepared samples was measured at 562 nm.

### Raman Imaging

A WITec Confocal Raman Microscope (WITec GmbH, Germany), supplied with an Ultra-High-Throughput Screening 300 spectrograph and a charge-coupled device (CCD) detector operated via WITec Control Software, was used to accomplish Raman imaging. All spectra were collected with a laser excitation of 532 nm and a 60× water immersion objective (Nikon Fluor; NA = 1). All spectra were collected in the range 0–3670 cm^–1^, with a spectral resolution of 3 cm^–1^. With this system, a spatial resolution of up to 300 nm is feasible. The mapped area was adjusted to the cell size. For the measured cells, a sampling density of 1 μm and an integration time of 0.1 s were used. The distribution of cellular components can be seen in Raman images by analyzing data from Raman imaging and based them on the integral intensity of specific bands, i.e., organic matter (2815–3015 cm^–1^), lipids (2840–2860 cm^–1^), RAR-BR (2214 cm^–1^), and cytochrome *c* (750 cm^–1^). From each sample, up to 20 cells were imaged.

### Data Analysis and Processing

Software from WITec GmbH called WITec Plus was used to analyze the Raman data. Cosmic ray removal (filter size: 4; dynamic factor: 4) and background subtraction (polynomial order: 3) were used as part of the preprocessing of the Raman spectra. *K*-means cluster analysis (KMC) was used to examine the prepared spectra in the ranges 2800–3050 cm^–1^ and 500–1800 cm^–1^. Average spectra for the cytoplasm, cell nucleus, region of the cytoplasm with a high content of lipids, and region of the cytoplasm with a high content of cytochrome *c* were obtained as a consequence of cluster analysis. By averaging the Raman spectra of 15–20 cells from each of the KMCA classes (cytoplasm, nucleic acids, lipids, and cytochrome *c*), average Raman spectra were created. The Raman spectra displayed here were normalized and cut using Bruker’s Opus software and are in the 500–3050 cm^–1^ range. Origin 2021 (OriginLab Corp.) was used for data presentation.

### Fluorescence Imaging

Prior to the fluorescence measurement, cells were treated with RAR-BR (concentration: 50 nM–400 nM, incubation time: 15–30 min), and then the medium was changed, and cells were incubated with MitoTracker Orange CMTMRos (100 nM, 30 min). Cells were imagined in a Live cell buffer. The fluorescence measurements of live HAEC cells were taken using an optical microscope (Olympus CKX53, Olympus DP74, Tokyo, Japan) equipped with an Olympus LCAch N 40 (NA = 0.55, iPC) objective (Olympus, Tokyo, Japan), high-resolution DP74 camera (Olympus, Tokyo, Japan), and CellSens software (Olympus, Tokyo, Japan). The fluorescence filter cube used for the RAR-BR observations had a band-pass excitation filter of 360–370 nm and a band-pass barrier filter of 420–460 nm. For the MitoTracker Orange CMTMRos, the filter cube was equipped with a 530–550 nm excitation bandpass filter and an ET 600/50 nm barrier bandpass filter.

### Data Management

Raw measurement data related to conducted research are available here 10.57903/UJ/VMMSDQ. Based on these data, the figures presented in this publication were created.

## Results and Discussion

### Synthesis of RAR-BR

**Figure 1:**
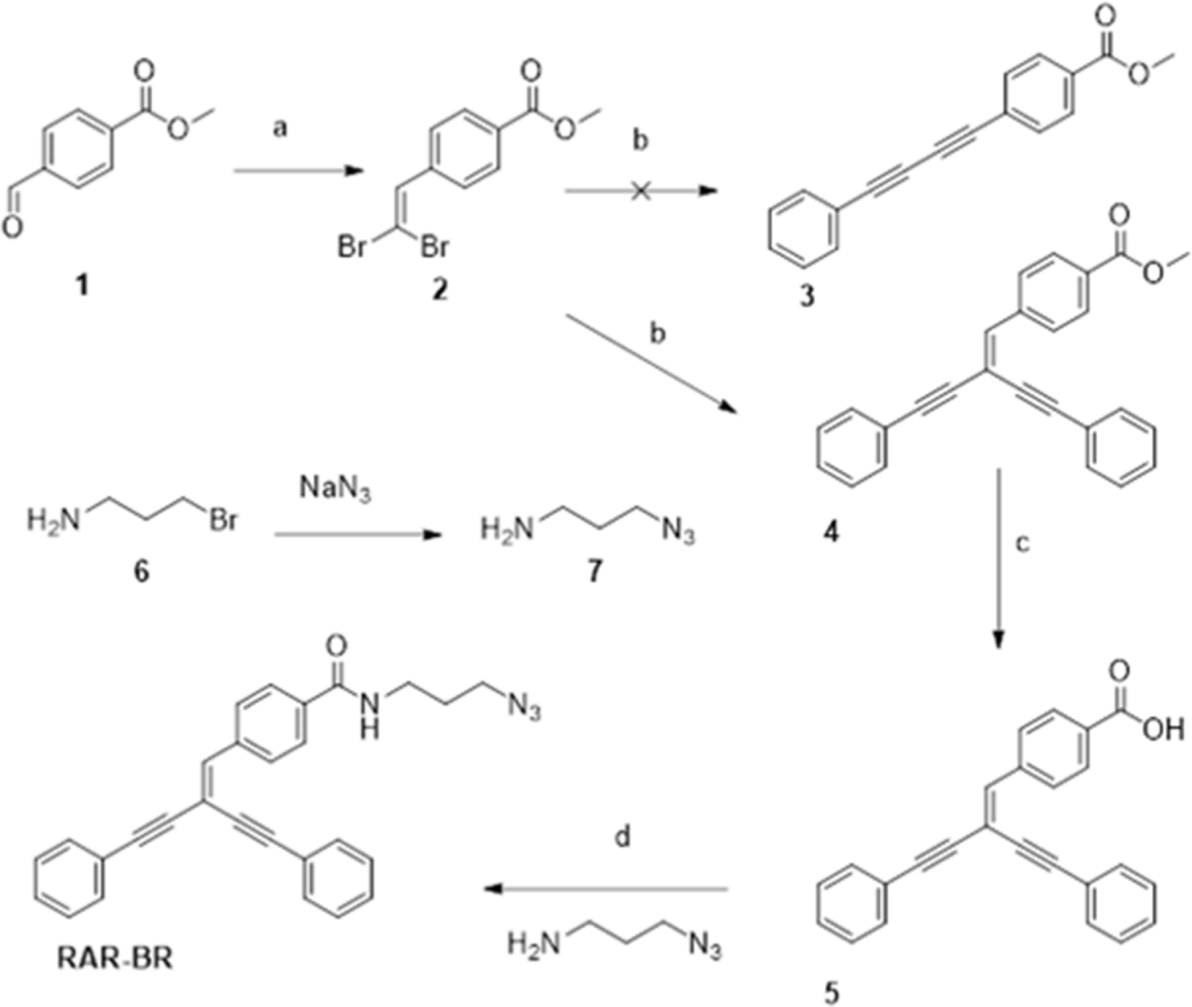
Synthesis of RAR-BR. (a) CBr_4_, PPh_3_, 0 °C to RT, 4 h. (b) PPh_3_, Pd_2_(dba)_3_, triethylamine, phenylacetylene, DMF, 85 °C, 4 h. (c) THF/H_2_O, LiOH monohydrate. RT, 3 h. (d) 3-azidopropan-1-amine, HATU, triethylamine, DMF, 60 °C, 16 h.

The synthesis of RAR-BR was carried out following a reported methodology (Figure 1).^51^ The 1,1-dibromo alkane was prepared by the reaction of CBr_4_ and triphenyl phosphine with methyl 4-formylbenzoate. However, the product obtained during the palladium-catalyzed reaction was not the 1,3-diyn **3**, as reported previously.^40^ Instead, we consistently observed the formation of the 1,1-diynyl-1-alkene **4** as the main product. (14 aromatic protons instead of 9, full NMR assignment can be found in the Supporting Information) and a signal corresponding to a tetra-substituted alkene. This is not a surprise since both products can be prepared in similar conditions.^52^ Further hydrolysis and amide coupling yielded RAR-BR. The structure was confirmed by NMR and mass spectrometry.

### Spectral Characterization of RAR-BR

**Figure 2:**
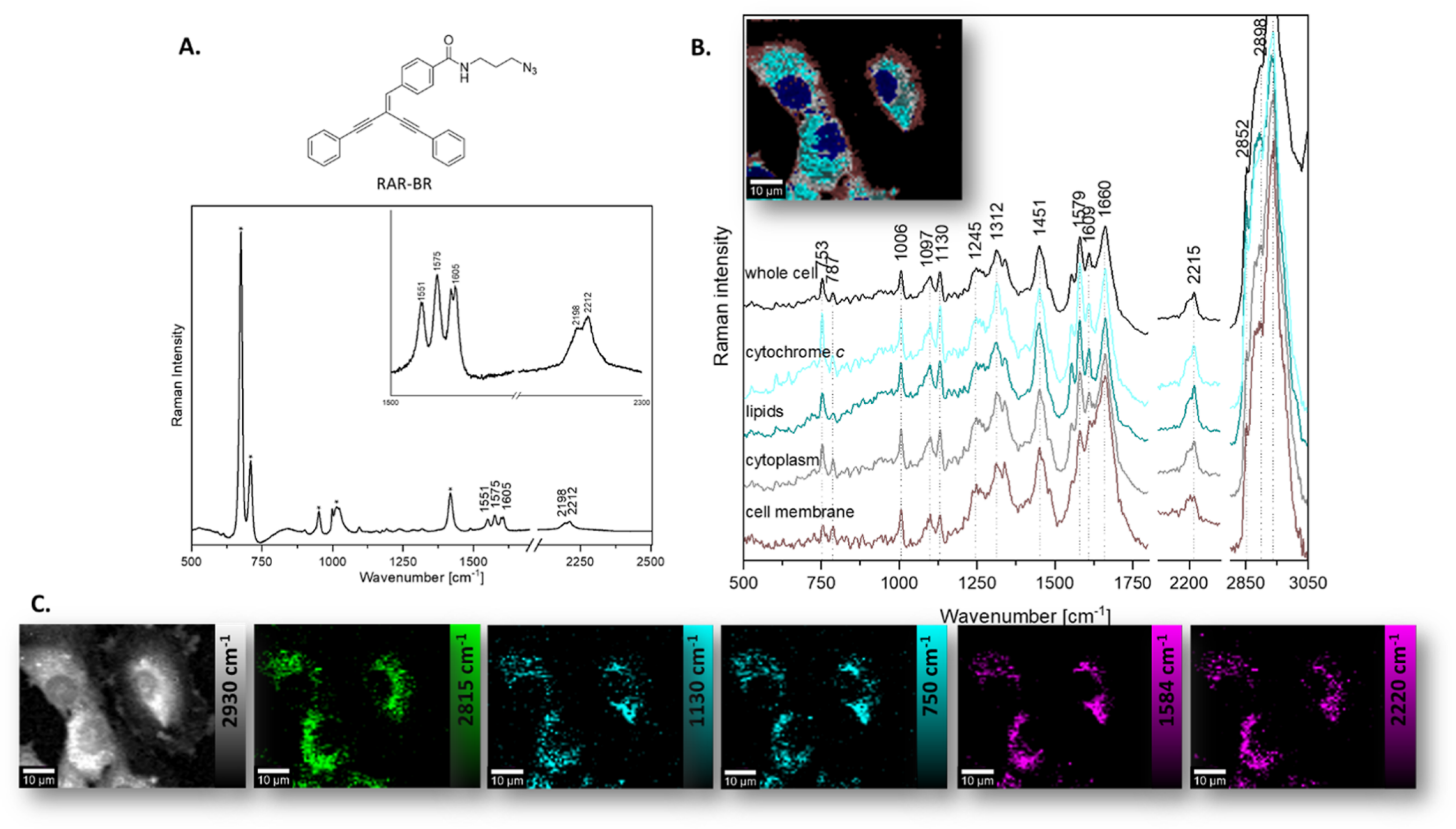
Spectral characterization of RAR-BR. (A) Structure and Raman spectrum of RAR-BR dissolved in DMSO. (B) KMCA-based average spectra of characteristic classes (cytoplasm region with high content of lipids (green), cytoplasm region with high content of cytochrome *c* (cyan), poor cytoplasm (gray), cell membrane (brown), and whole cell (black)) and cluster map (colors for each class are the same as for spectra). Raman. (C) Raman images of live HAEC incubated with RAR-BR (50 nM, 15 min) obtained by the integration of Raman bands over the selected Raman bands at 2930 cm^–1^ (organic matter), 2850 cm^–1^ (lipids), 1130 cm^–1^ (cytochrome *c*), 752 cm^–1^ (cytochrome *c*), 1584 cm^–1^ (RAR-BR), and 2218 cm^–1^ (RAR-BR). Scale bar: 10 μm.

Here, we report on a new Rp that can be used for specific and selective detection and visualization of mitochondria and, prospectively, for tracking their activity. The Raman spectrum of RAR-BR shows characteristic bands in the silent spectral region, in addition to the fingerprint range (Figure 2A). An intense band with two maxima at 2212 and 2198 cm^–1^, assigned to two nonconjugated triple bonds, is observed. Additionally, three characteristic bands can be distinguished at 1605 cm^–1^ (C=O band), 1570 cm^–1^ (−C(=O)–N band, aromatic ring), and 1546 cm^–1^ (aromatic ring). This spectral profile is very unique, so RAR-BR offers a very clear and discrete marker. To study the uptake and cellular localization of the probe, ECs were incubated with RAR-BR for 15 min at 50 nM. By employing KMCA, it was possible to isolate individual cell classes, i.e., cytoplasm with a high content of lipids, cytoplasm with a high content of cytochrome *c* (mitochondria), and cytoplasm with a low content of lipids and cytochrome *c*, cell membrane, and cell nucleus. Figure 2B, C shows spectra and images obtained with KMCA, where the individual most relevant cell classes are seen. The analysis led to the identification of areas of accumulation of the tested Rp. RAR-BR accumulates mainly in the cytoplasm with a high content of cytochrome *c*. However, the compound due to lipophilicity can also be found to some extent in the lipid-rich cytoplasm and also in the cell membrane.

Nonspecific accumulation is an inherent problem with mitochondria-targeting compounds, leading to a large background from off-target sites in imaging. Therefore, it is critical to properly select experimental conditions in order to avoid nonspecific Rp accumulation.

### High Sensitivity of RAR-BR to Probe Mitochondria

**Figure 3:**
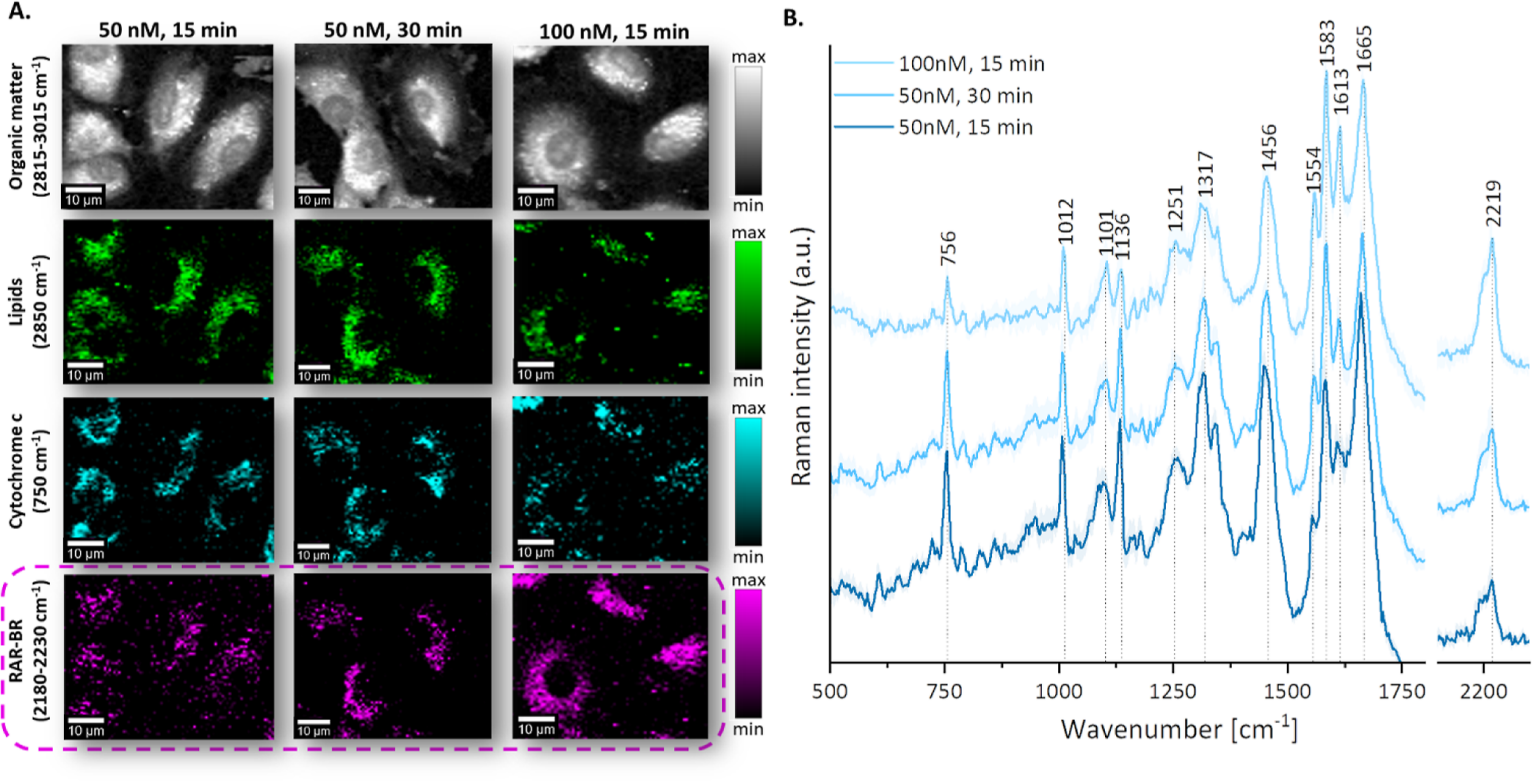
Raman imaging of live HAEC cells incubated with RAR-BR. (A) Selected experimental conditions: 50 nM, 15 and 30 min, 100 nM, 15 min, obtained by the integration of Raman bands over the selected Raman bands at 2930 cm^–1^ (organic matter), 2850 cm^–1^ (lipids), 750 cm^–1^ (cytochrome *c*), and 2218 cm^–1^ (RAR-BR). (B) Average Raman spectra (±SD, standard deviation) of the whole cytoplasm region (without nucleus) in fingerprint and silent regions for selected conditions. Scale bar: 10 μm.

**Figure 4:**
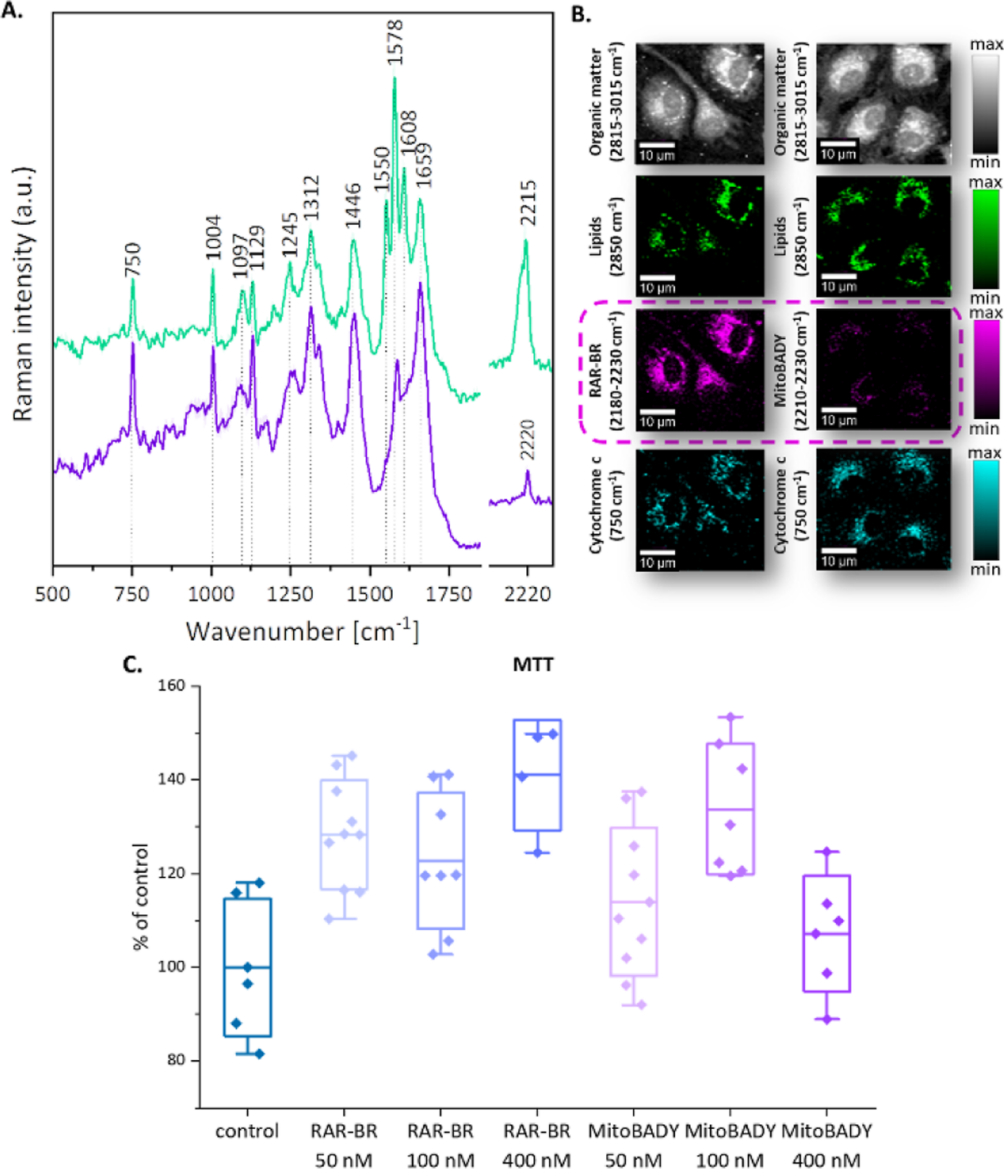
Raman images of live HAEC cells incubated with RAR-BR and MitoBADY. (A) Raman images obtained by the integration of Raman bands over the selected Raman bands at 2930 cm^–1^ (organic matter), 2850 cm^–1^ (lipids), 752 cm^–1^ (cytochrome *c*), 2214 cm^–1^ (RAR-BR), and KMCA image. (B) Average Raman spectra (±SD, standard deviation) of whole cytoplasm without nucleus for cell incubated with RAR-BR (gray) and MitoBADY (red). Incubation time: 30 min, concentration: 400 nM. (C) Cell viability test established by the MTT test after 30 min incubation with BAR-BR and MitoBADY at concentrations 50, 100, and 400 nM (*x-axis*-axis: conditions, *y-axis*-axis: the mean percentage of surviving cells relative to the number of untreated cells). Results are presented as box plots (mean ± SD, standard deviation, whiskers indicate minimum/maximum, and line indicates the mean value). Scale bar: 10 μm.

In order to determine the optimal experimental conditions for RAR-BR imaging of mitochondria, several conditions were iterated. Here, we will discuss conducted experiments to determine the lowest concentration and incubation time that allowed Rp to be detected in a selective and specific way in mitochondria. To ensure strong mitochondrial activity, concentrations of RAR-BR from 50 nM to 5 μM were tested, with an incubation time of 30 min (the time was selected based on the previous experience with MitoBADY testing). Based on KMCA, the lowest concentration of RAR-BR that can be used for imaging was found to be 50 nM (Figure 3). After the concentration was determined, the next step was to select the appropriate incubation time. Here, too, a wide range of time between 5 and 60 min was tested. The analysis shows that when the cells are incubated with 50 nM of the compound, a time of 30 min (Figure 3) yielded satisfactory colocalization with cytochrome *c*. It is also worth noting that in the case of 100 nM, this time is reduced to 15 min, and the results are very similar (Figure 3). Both higher concentration and prolonged incubation time resulted in nonspecific accumulation of the Rp in the lipidic structures in the cell. This is not surprising; a similar problem occurs in the case of MitoBADY, however, here we can use a much lower concentration with a shorter incubation time. Moreover, in the case of MitoBADY, tendency to weak accumulation at low concentrations in ECs were observed, which is not a problem when using RAR-BR. This may be related to the lower ability of the compound to cross EC membranes compared with the new Rp. Comparing RAR-BR (100 nM, 30 min) with commercially available MitoBADY (100 nM, 30 min) (Figure 4A, B), the great potential of the new Rp can be seen. Using the same experimental parameters for Raman imaging of cells incubated with Rp, i.e., the concentration of Rp and time of incubation, the intensity of the RAR-BR signal is several times higher than that for MitoBADY. So, a lower concentration and time of incubation can be used for RAR-BR to achieve a similar action to MitoBADY. This is due to the fact that RAR-BR contains two equiv of alkyl, which is associated with a higher band intensity in the silent region of the Raman spectrum.

To estimate the effects of the studied compounds on cell viability, the thiazolyl blue tetrazolium bromide (MTT) assay was used, which is one of the most common tests to indicate succinate dehydrogenase (complex II) activity in the respiratory electron transport chain. The results were expressed as the mean percentage of surviving cells relative to the number of control cells (untreated cells) as 100%. Higher values of the absorbance in the experimental group in comparison to the control group indicate that the tested compounds, at the concentrations used in the experiments, did not cause a decrease in cell viability (Figure 4C).

**Figure 5:**
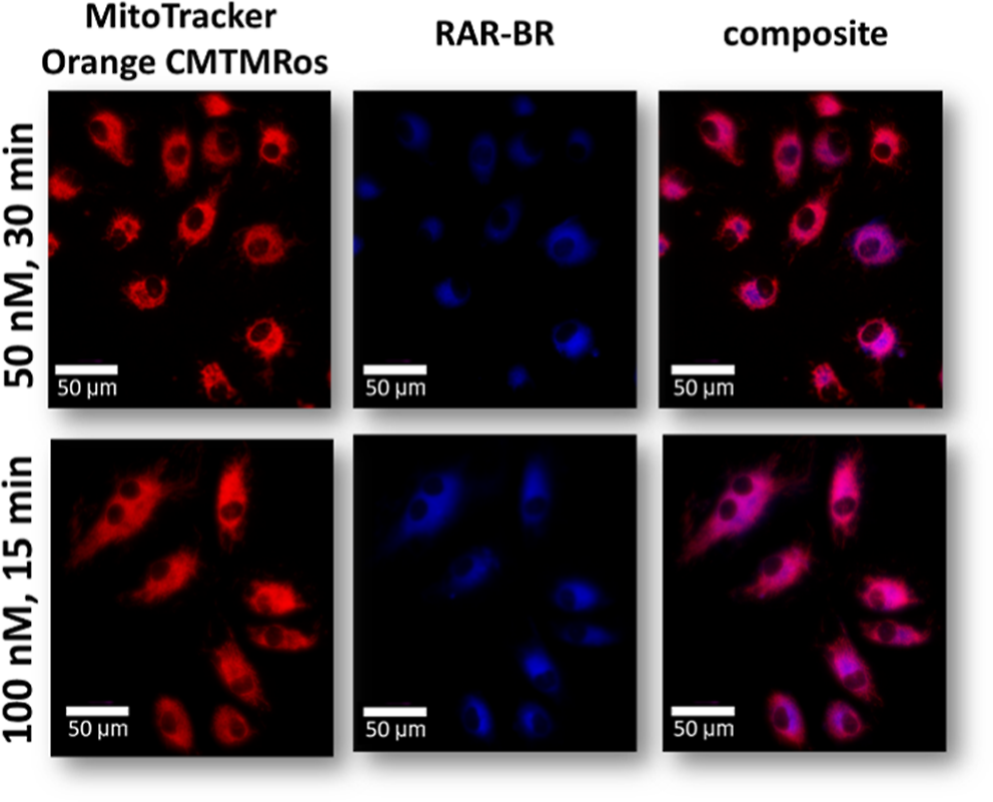
Fluorescence images of mitochondria in live HAEC cells stained with MitoTracker Orange CMTMRos and RAR-BR. Images were collected with a 40× objective and an Olympus Scan∧R system. At the top, the incubation time and concentration for both probes are given. Scale bar: 10 μm.

Additionally, to confirm that the RAR-BR probe shows good colocalization with mitochondria, fluorescence imaging was performed (Figures 5 and S14). Cells were incubated with the RAR-BR probe at various concentrations from 50 to 400 nM (em: 423 nm) and stained by MitoTracker Orange CMTMRos (em: 576 nm). Images were collected with a 40× objective and Olympus Scan∧R system. Figure 3 shows fluorescent images of mitochondria in live HAEC cells. Comparison of fluorescence images for RAR-BR and MitoTracker shows very good colocalization at lower concentrations. That confirms that RAR-BR is a good probe for detecting mitochondria in ECs using Raman imaging. Figure S15 demonstrates the fluorescence intensity profiles for MitoTracker Orange CMTMRos and the RAR-BR Rp. A very similar pattern for both dyes confirms the thesis of their correlation in the cell.

### Mechanism of Action of RAR-BR and Comparison with MitoBADY

As shown above, RAR-BR has an affinity for mitochondria, and several hypotheses have been put forward to explain its mechanism of action. The first hypothesis is that RAR-BR can attach to the mitochondrial membrane with a negative potential due to its positively charged group. The positively charged group is a characteristic structural block of other mitochondria targeting probes.^16^ The azide at the end of the amide part in the cell environment can be reduced, creating a positive charge and allowing Rp to be bonded to the mitochondrial membrane. Our second hypothesis is that the RAR-BR interacts with a receptor on the mitochondrial membrane and forms a covalent bond with a specific protein. This mechanism of action has been previously reported;^12^ however, it can cause inhibition of mitochondrial respiratory activity with long incubation and high concentration.^12^

**Figure 6:**
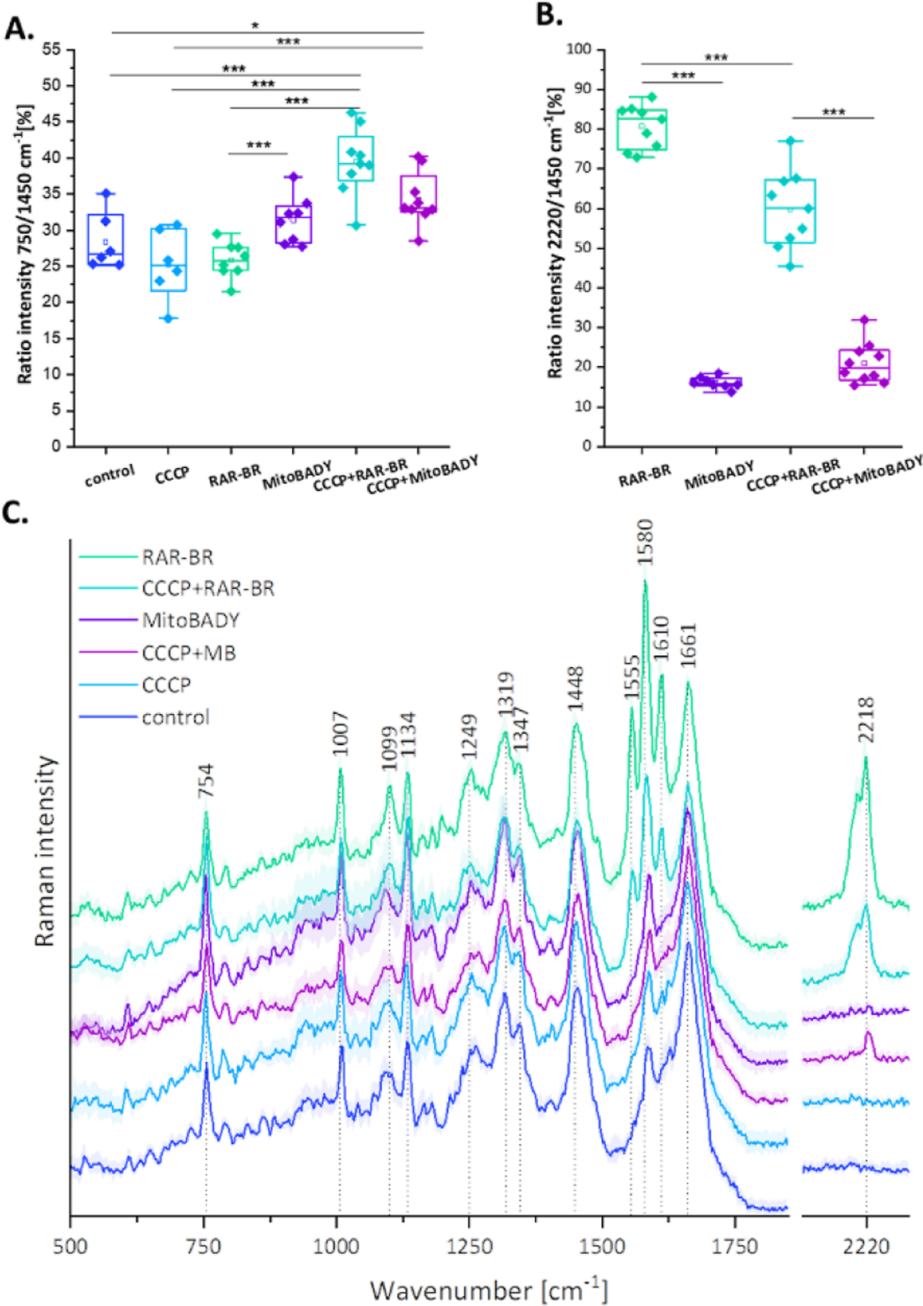
Raman imaging of live HAEC cells incubated with RAR-BR and CCCP. (A) Integral intensity ratio of bands at 750/1450 cm^–1^ and (B) 2220/1450 cm^–1^. (C) Average Raman spectra (±SD, standard deviation) of the whole cytoplasm region (without nucleus) for control, CCCP (0.7 μM, 15 min), RAR-BR (100 nM, 30 min), CCCP (0.7 μM, 15 min) + RAR-BR (100 nM, 30 min), MitoBADY (100 nM, 30 min), and CCCP (0.7 μM, 15 min) + MitoBADY (100 nM, 30 min). Results are presented as box plots (mean ± SD, standard deviation, whiskers indicate minimum/maximum, and line indicates the mean value), *p < 0.05, ***p < 0.001.

In order to verify the above hypotheses, first a depolarization of the mitochondrial membrane was conducted using an uncoupling agent, i.e., carbonyl cyanide *m*-chlorophenyl hydrazone (CCCP). CCCP is a protonophore that is widely used to investigate the function of mitochondria. CCCP is a potent uncoupler of mitochondrial OXPHOS. It disrupts ATP synthesis by transporting protons across the mitochondrial inner membrane, interfering with the proton gradient.^53–55^ The use of CCCP (0.7 μM, 15 min) causes a decrease in the mitochondrial membrane potential, which is observed from a reduced band intensity ratio of 750/1450 cm^–1^ in comparison to that of the control. Taking into account the obtained results, it can be postulated that the tested Rp forms covalent bonds with mitochondrial proteins, which slightly changes the mitochondrial activity [decrease in the ratio of 750/1450 cm^–1^, as in the case of CCCP (Figure 6A,C)]. The reduced mitochondrial activity by CCCP followed by RAR-BR results in less accumulation of the compound in the mitochondria (lower ratio of 2220/1450 cm^–1^) compared to samples with RAR-BR alone (Figure 6B,C).

It has already been reported that MitoBADY has quite an intense Raman band in the silent region in comparison to another Rp 5-ethynyl-20-deoxyuridin (EdU), i.e., approximately 25 times stronger.^43^ Here, we compared the Raman activity of RAR-BR and MitoBADY by measuring the spectra of the sample solutions of the same concentration (Figure S16). It was noticed that in the case of MitoBADY a small band can be observed, while RAR-BR does not give a signal in the quiet range under these conditions. The lack of signal results from an increase in background and significant fluorescence (the color of the solution is yellowish). We can thus conclude that the primary effect of RAR-BR is that of superior mitochondrial localization rather than that of improved Raman cross-section.

Comparing the structure of RAR-BR with MitoBADY, in MitoBADY the mitochondrial targeting moiety is a triphenylphosphonium cation (TPP^+^). Lipophilic cations accumulate inside mitochondria according to the Nernst equation. In our previous work,^56^ we showed that preincubation of cells with an uncoupler causes an increased amount of MitoBADY entry into mitochondria, which suggests that the action of RAR-BR and MitoBADY is different.

## Conclusions

In this manuscript, we report on a new Rp that can be used for specific and selective detection and visualization of mitochondria. The spectral profile of RAR-BR is very unique; characteristic bands in the silent spectral region, in addition to the fingerprint range, can be observed, so Rp offers a very clear and discrete marker. We optimized the conditions for the use of RAR-BR in live ECs. The analysis shows that when the cells are incubated with 50 nM compound, a time of 30 min yielded satisfactory colocalization with cytochrome *c*. It is also worth noting that in the case of 100 nM, this time is reduced to 15 min, and the results are very similar. Both higher concentration and prolonged incubation time resulted in nonspecific accumulation of the Rp in the lipidic structures in the cell. The comparison of RAR-BR with commercially available Rp targeting mitochondria MitoBADY showed that lower concentration and time of incubation can be used for RAR-BR to achieve a similar action to MitoBADY since RAR-BR contains two equiv of alkyl, which is associated with a higher band intensity in the silent region of Raman spectrum which is a key advantage of the new Rp. We also examined the mechanism of action of RAR-BR and postulate that our Rp forms covalent bonds with mitochondrial proteins, indicating that the actions of RAR-BR and MitoBADY are different.

We believe that this new Rp has the prospect of being used to track mitochondrial changes and activity in the future.

## Supporting Information Available

Synthesis of RAR-BR, ^1^H NMR spectra, ^13^C NMR spectra, and mass spectra of RAR-BR (PDF)

## Abbreviations

3DSIM: three-dimensional structured illumination microscopy
ATP: adenosine triphosphate
BADY: bisarylbutadiyne
BG: background subtraction
CCCP: carbonyl cyanide *m*-chlorophenyl hydrazone
CRR: cosmic ray removal
EC: endothelial cells
EdU: 5-ethynyl-20-deoxyuridin
EM: electron microscopy
FM: fluorescence microscopy
FR: free radical
HAEC: human aortic endothelial cells
KMC: *k*-means cluster
MTT: thiazolyl blue tetrazolium bromide
OXPHOS: oxidative phosphorylation
RAR-BR: *N*-(3-azidopropyl)-4-(4-phenyl-2-(phenylethynyl)but-1-en-3-yn-1-yl)benzamide
RM: Raman microscopy
ROS: reactive oxygen species
Rp: Raman probe
SMLM: single-molecule localization microscopy
STED: stimulated emission depletion
TPP: triphenylphosphonium cation
