# Supplementary material for: New Highly Sensitive and Specific Raman Probe for Live Cell Imaging of Mitochondrial Function
Source: ACS Sens. 2024 Feb 9;9(2):995–1003. doi: 10.1021/acssensors.3c02576 (PMC10897933; doi:10.1021/acssensors.3c02576)
Supplement: Supplementary file 1 — se3c02576_si_001.pdf [file se3c02576_si_001.pdf]

A new highly sensitive and specific Raman probe for live cell imaging of mitochondrial function

Anna Pieczara[1,2], Ruben Arturo Arellano Reyes[4], Tia Keyes[4], Patrycja Dawiec[2,3], Malgorzata Baranska[1,3,*]

[1] *Jagiellonian Centre for Experimental Therapeutics (JCET), Jagiellonian University, 14 Bobrzynskiego Str., 30-348 Kra-kow, Poland*

[2] *Jagiellonian University in Kraków, Doctoral School of Exact and Natural Sciences, 11 Lojasiewicza St., Krakow, Poland*

[3] *Faculty of Chemistry, Jagiellonian University, 2 Gronostajowa Str., 30-387 Krakow, Poland*

[4] *School of Chemical Sciences, Dublin City University, 592, 628 Collins Ave Ext, Whitehall Dublin 9, D09 E432, Dublin, Ireland*

[*] Corresponding author: m.baranska@uj.edu.pl

Keywords: mitochondrial membrane; Raman probe; RAR-BR; CCCP; spontaneous and stimulated Raman microscopy

Methyl 4-(2,2-dibromovinyl)benzoate (2). In a 100 mL round-bottom flask, carbon tetrabromide (2.02 g, 6.09mmol, 1 eq.) was dissolved in DCM and the solution cooled to 0°C in an ice-water bath. Triphenylphosphine (3.20 g, 12.18 mmol, 2 eq.) was added slowly and the solution stirred for 20 minutes at that temperature. Methyl-4-formylbenzoate dissolved in 20 mL of DCM was then added slowly to the reaction mixture. The ice-water bath was removed, and the solution was stirred for 4 h and allowed to reach room temperature. The solvent was evaporated and the product purified by column chromatography using silica gel and cyclohexane:ethyl acetate (8:2) to afford **2** as a white solid in 74% yield. ^{1}H-NMR (600MHz, $CDCl_3$) δ ppm 8.03 (d, 3J = 8.46, 2H), 7.59 (d, 3J = 8.28, 2H), 7.52 (s, 1H), 3.92, (s, 3H).

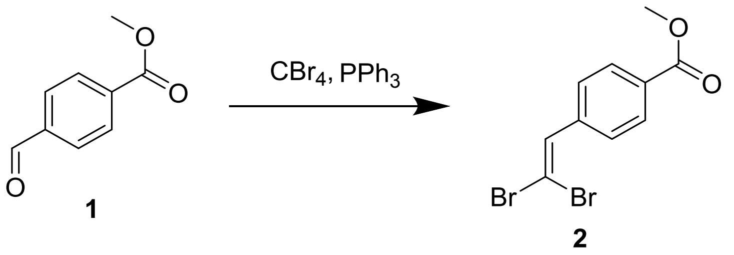

Figure S1. Synthesis of Methyl 4-(2,2-dibromovinyl)benzoate (2).

Methyl 4-(4-phenyl-2-(phenylethynyl)but-1-en-3-yn-1-yl)benzoate (4). In a sealed tube, 856 mg of **1** (2.68 mmol) was dissolved in 3 mL of dry DMF, phenylacetlyne (441 µL, 4.01 mmol, 1.5 eq.), triethylamine (1.12 mL, 8.08 mmol, 3 eq.), triphenylphosphine (28 mg, 0.107 mmol, 0.04 eq.), and $Pd_2(dba)_3$ (24.5 mg, 0.026 mmol, 0.01 eq.) were added. The mixture was stirred at 85 °C for 4 hours. After cooling to room temperature, the solution was diluted with ethyl acetate and washed with diluted hydrochloric acid (1M, 3 x 30 mL) and then with sodium hydroxide (1M, 3x 30 mL). The combined organic fractions were dried over magnesium sulphate and the solvent evaporate under vacuum. 3 portions of 30 mL of heptane were added to fully remove DMF. The product was purified by column chromatography using silica gel and EtAco:Cyclohexane (8:2 V/V) to afford **3** as a yellow solid in 67% yield. ^{1}H-NMR (600MHz, $CDCl_3$) δ ppm 8.06 (d, ^3J = 8.41, 2H), 8.00 (d, ^3J = 8.41, 2H), 7.58-7.52 (m, 4H), 7.41-7.37 (m, 3H), 7.37-7.33 (m, 3H), 7.18 (s, 1H), 3.93 (s, 3H). ^{13}C-NMR (150 MHz, $CDCl_3$) δ ppm 166.6, 141.5, 139.9, 131.8, 131.7, 130.0, 129.6, 129.1, 128.8, 128.7, 128.5, 128.3, 122.6, 122.5, 105.8, 95.6, 89.4, 88.9, 86.6, 52.1.

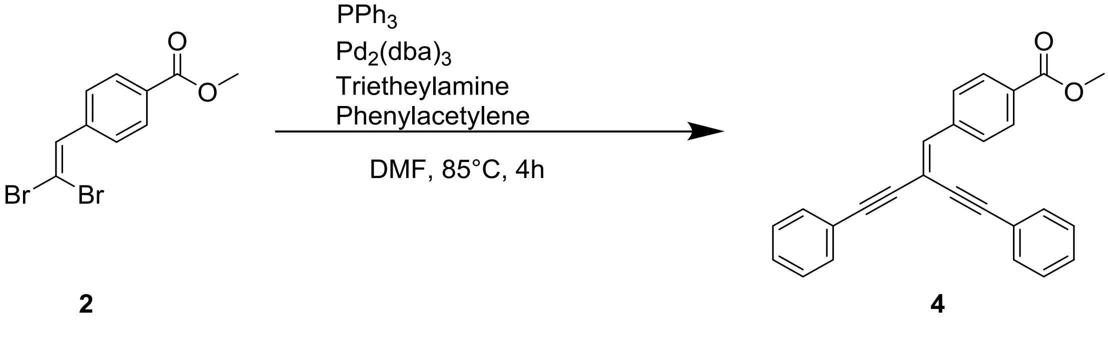

Figure S2. Synthesis of Methyl 4-(4-phenyl-2-(phenylethynyl)but-1-en-3-yn-1-yl)benzoate (4).

4-(4-phenyl-2-(phenylethynyl)but-1-en-3-yn-1-yl)benzoic acid (5). In a 50 mL round-bottom flask, 338 mg of 3 (0.932 mmol) were dissolved in 10 mL of THF. Lithium hydroxide

monohydrate (78.27 mg, 1.87 mmol, 2 eq.) was dissolved in 1 mL of water and was added to the flask. The reaction was stirred at room temperature for 3 hours. The solution was acidified with HCl until pH 3 and poured onto ice. The product precipitated as a light-yellow solid and was filtered under vacuum. Intermediate **3** was obtained in quantitative yield and was pure enough to be used without purificaiton in the next step. ^{1}H-NMR (600MHz, DMSO-d$_6$) δ ppm 13.09 (s, 1H), 8.10 (d, ^{3}J = 8.47, 2H), 8.03 (d, ^{3}J = 8.47, 2H), 7.66-7.622 (m, 2H), 7.60-7.56 (m, 2H), 7.51-7.43(m, 7H).

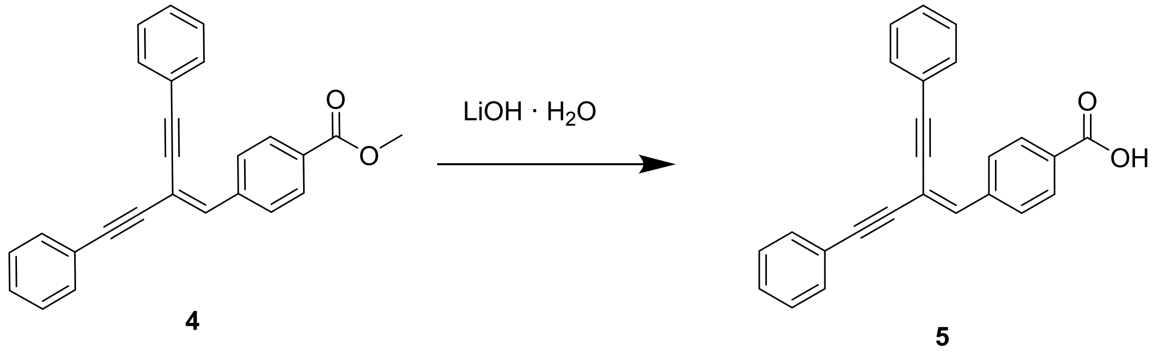

Figure S3. Synthesis of 4-(4-phenyl-2-(phenylethynyl)but-1-en-3-yn-1-yl)benzoic acid (5).

N-(3-azidopropyl)-4-(4-phenyl-2-(phenylethynyl)but-1-en-3-yn-1-yl)benzamide (RAR-BR). In a 25 mL Schlenk flask, 147 mg of **5** (0.421 mmol), 241 mg of HATU (0.532 mmol, 1.5 eq.), 106 mg of 3-azidopropan-1-amine (1.05 mmol, 2.5 eq.) and three drops of triethylamine were dissolved in 3 mL of DMF. The mixture was heated at 60°C for 16h under inert atmosphere. The solvent was evaporated with heptane under vacuum and the product was purified by column chromatography using silica gel and cyclohexane:EtAcO (6:4) to afford a yellow solid in 71 %yield. ^{1}H-NMR (600MHz, CDCl$_3$) δ ppm 8.00 (d, ^{3}J = 8.41, 2H), 7.79 (d, ^{3}J = 8.41, 2H), 7.57-7.52 (m, 4H), 7.40-7.37 (m, 3H), 7.37-7.33 (m, 3H), 7.17 (s, 1H), 6.40 (s, 1H), 3.57(q, ^{3}J = 6.41, 2H), 3.46 (t, ^{3}J = 6.46, 2H), 1.92 (p, ^{3}J =6.51, 2H). ^{13}C-NMR (150 MHz, CDCl$_3$) δ ppm 167.1, 141.6, 138.9, 134.3, 131.9, 131.8, 129.2, 129.2, 128.8, 128.7, 128.5, 127.1, 122.8, 122.7, 105.5, 95.6, 89.5, 89.0, 86.7, 49.8, 38.0, 28.9. HR-MS (ESI) m/z: calculated for $C_{28}H_{23}N_4O$ 431.1871 found 431.1816.

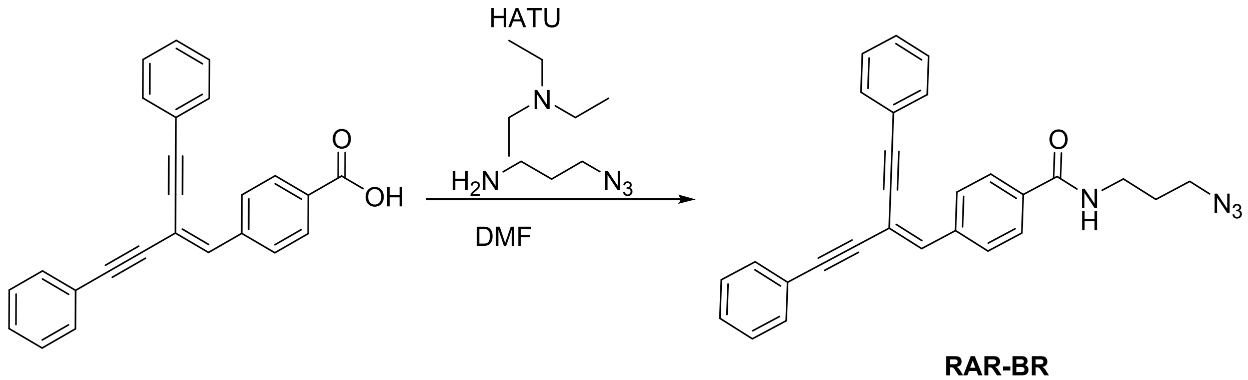

Figure S4. Synthesis of N-(3-azidopropyl)-4-(4-phenyl-2-(phenylethynyl)but-1-en-3-yn-1-yl)benzamide (RAR-BR).

3-azidopropan-1-amine (7). In a 100 mL round-bottom flask, 3-bromopropylamine hydrobromide (2 g, 9.14 mmol) was dissolved in 10 mL of water, sodium azide (2.97 g, 45.68 mmol, 5 eq.) was dissolved in 15 mL of water and added to the flask. The resulting solution was refluxed for 16h. The solution was allowed to cool to room temperature, 30 mL of a 1M potassium hydroxide solution was added and the solution was then transferred to an extraction funnel and extracted with diethyl ether (3 x 30 mL). The combined organic layers were dried over magnesium sulphate and the solvent evaporated to afford 1 as a viscous yellow oil in 89% yield. ^{1}H-NMR (600 MHz, $CDCl_3$) δ ppm 3.37 (t, ^3J = 6.71, 2H), 2.80 (t, ^3J = 6.81, 2H), 1.72 (p, ^3J = 6.76, 2H), 1.17 (br, 2H).

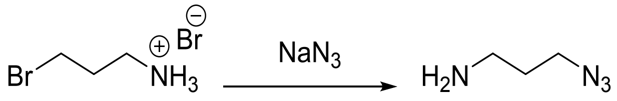

Figure S5. Synthesis of 3-azidopropan-1-amine (7).

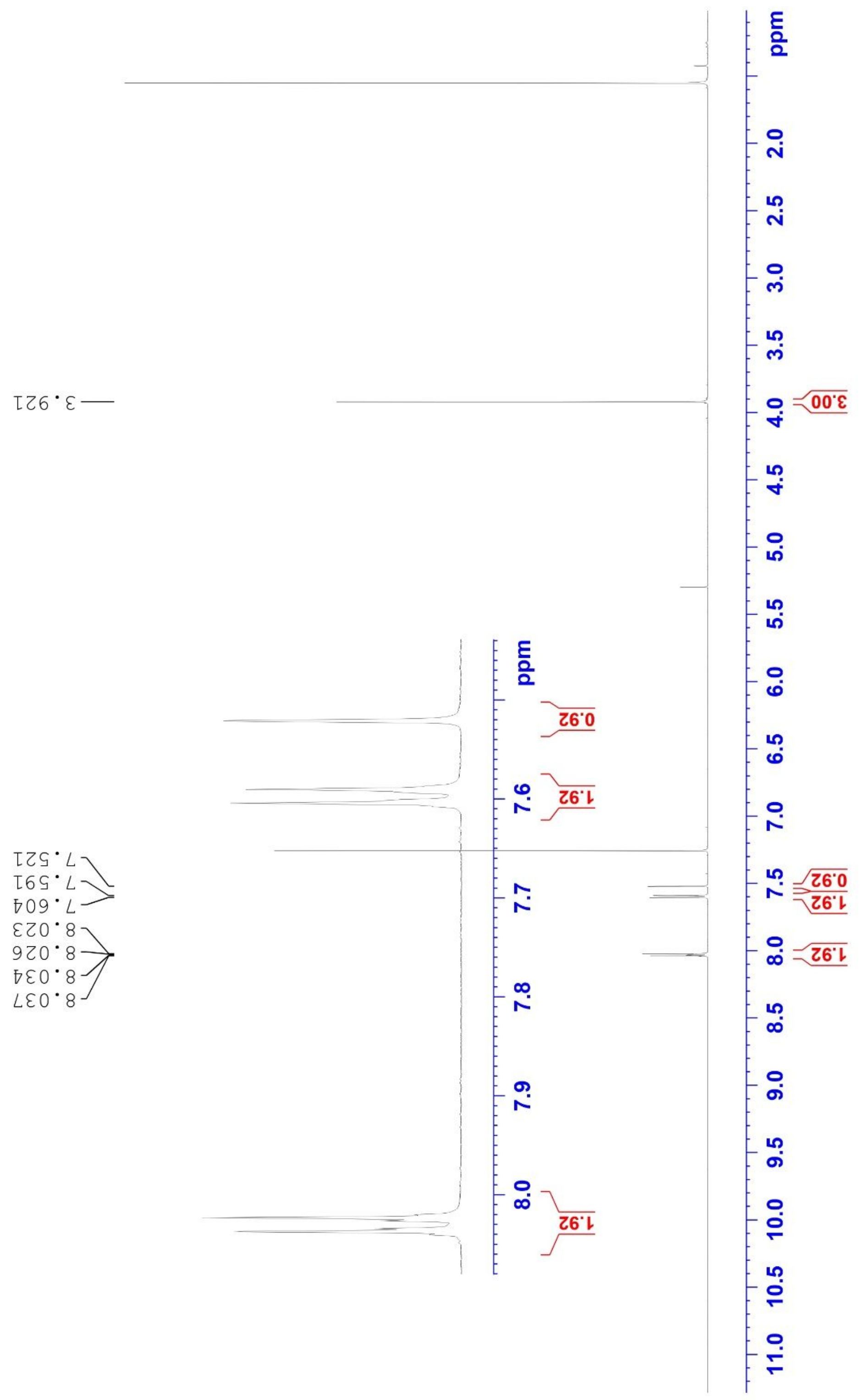

Figure S6. ^{1}H-NMR spectrum of Methyl 4-(2,2-dibromovinyl)benzoate **(2).** (600 MHz, $CDCl_3$).

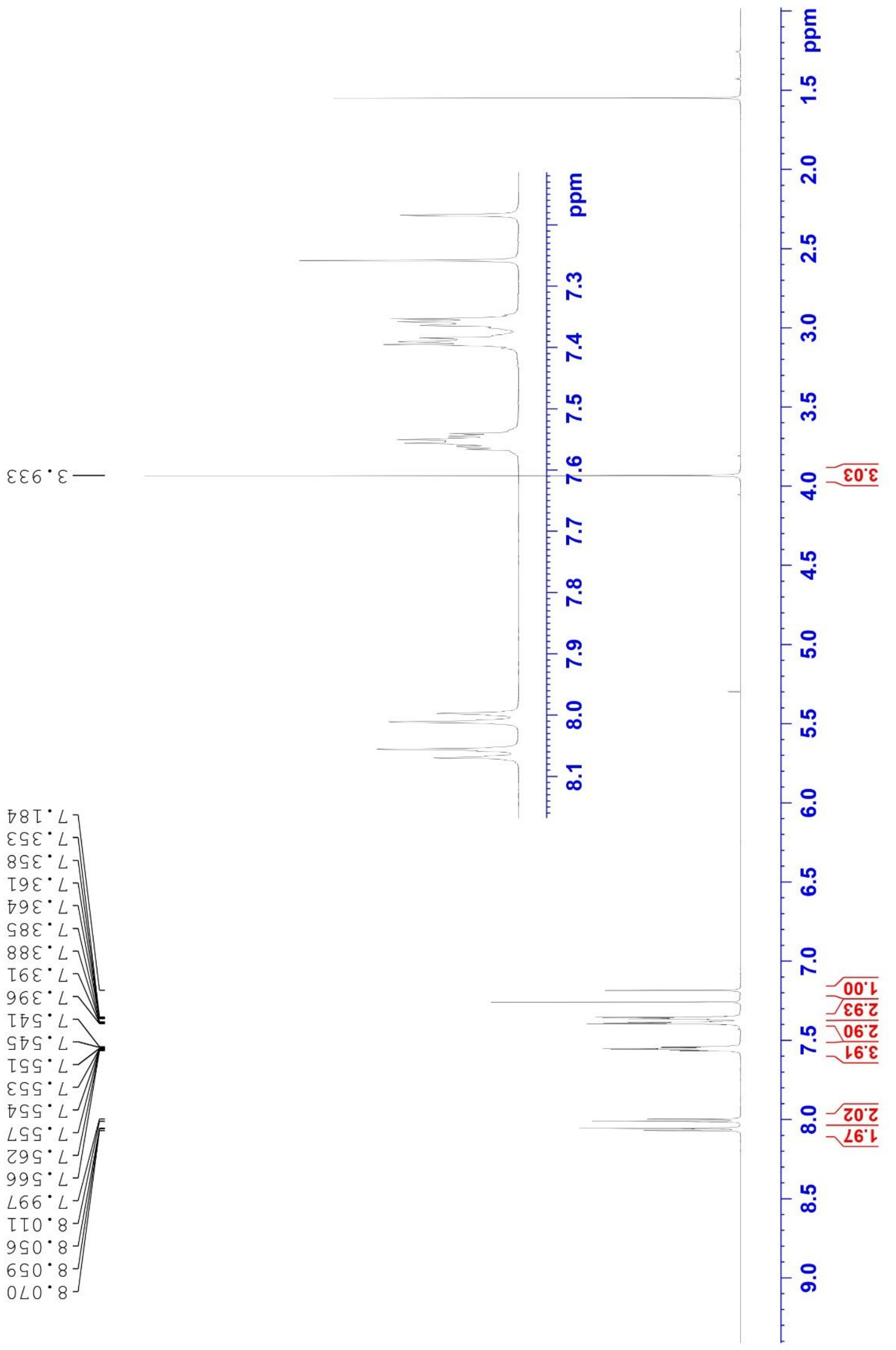

Figure S7. ^{1}H-NMR spectrum of methyl 4-(4-phenyl-2-(phenylethynyl)but-1-en-3-yn-1-yl)benzoate (4). (600 MHz, $CDCl_3$).

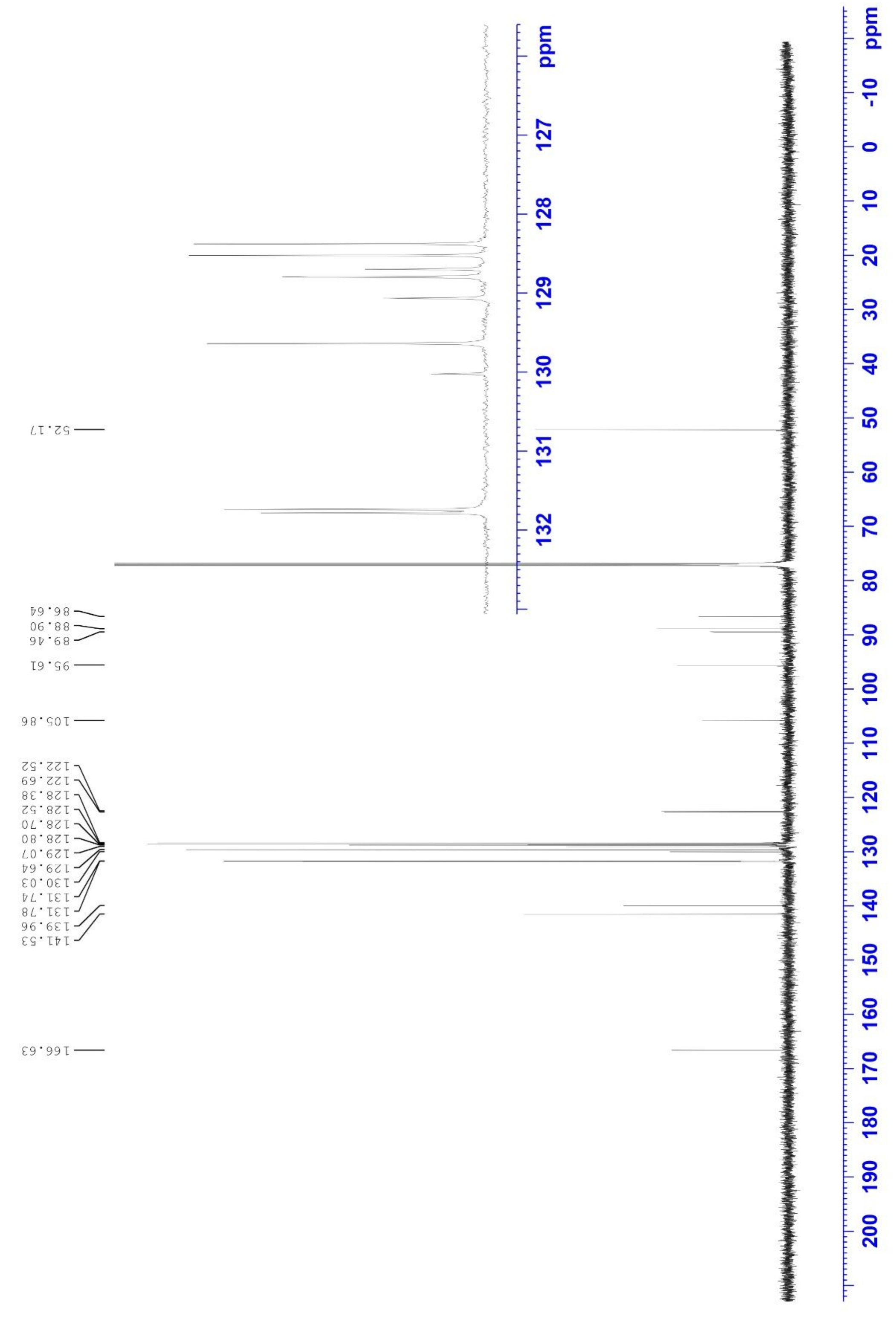

Figure S8. ^{13}C-NMR spectrum of methyl 4-(4-phenyl-2-(phenylethynyl)but-1-en-3-yn-1-yl)benzoate (4). (150 MHz, $CDCl_3$).

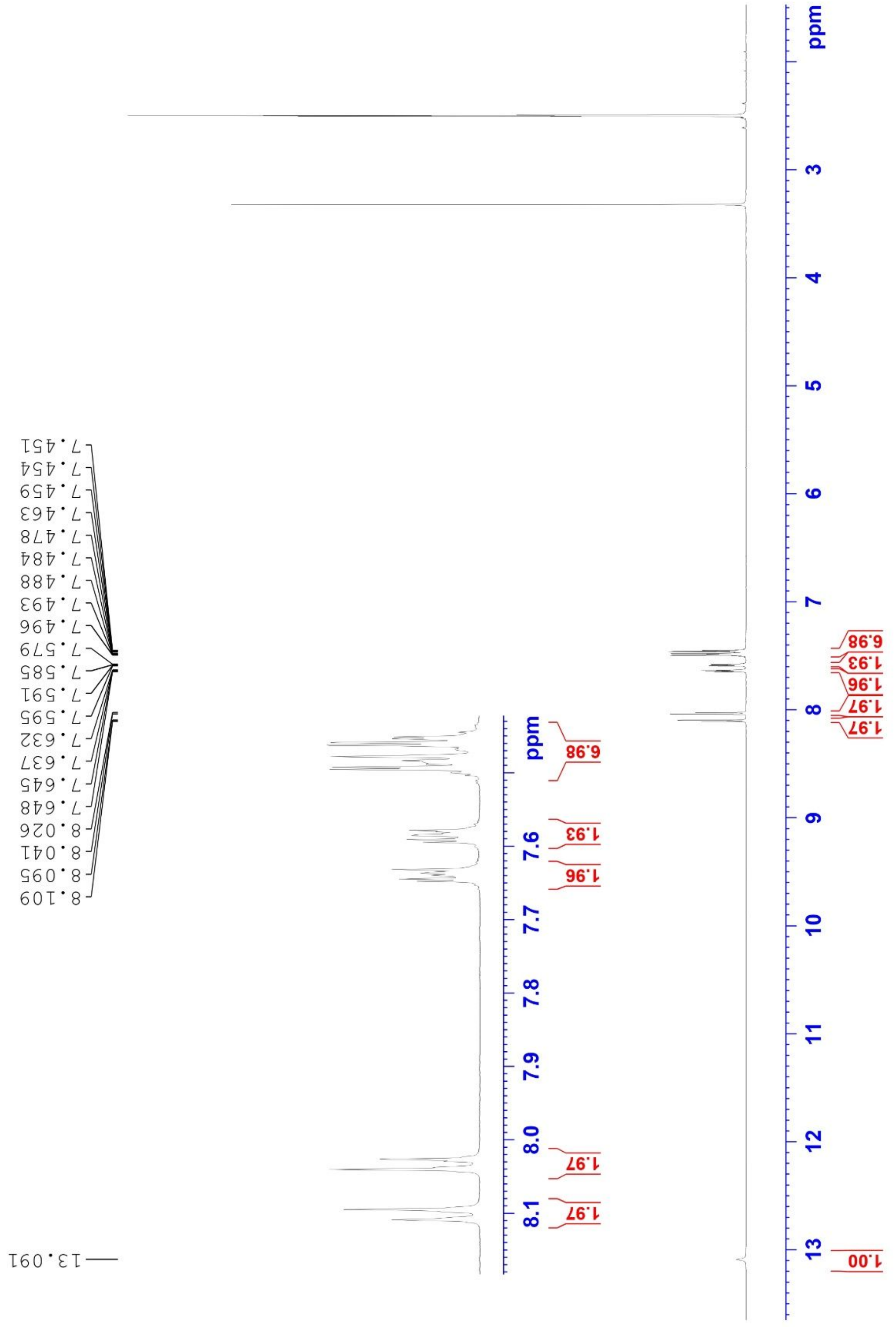

Figure S9. ^{1}H-NMR spectrum of 4-(4-phenyl-2-(phenylethynyl)but-1-en-3-yn-1-yl)benzoic acid (5). (600 MHz, DMSO-d_6).

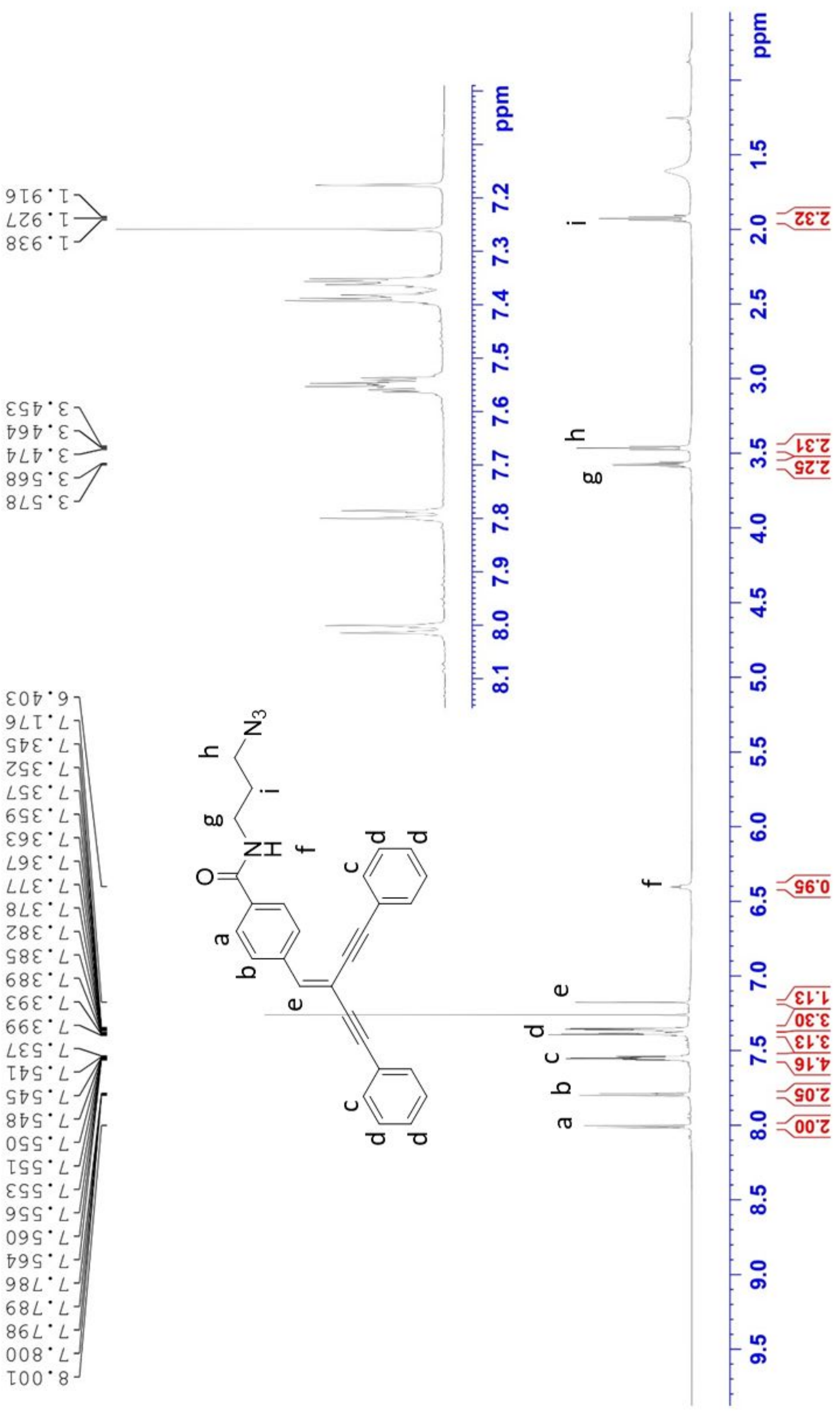

Figure S10. ^{1}H-NMR spectrum of RAR-BR. (600 MHz, $CDCl_3$).

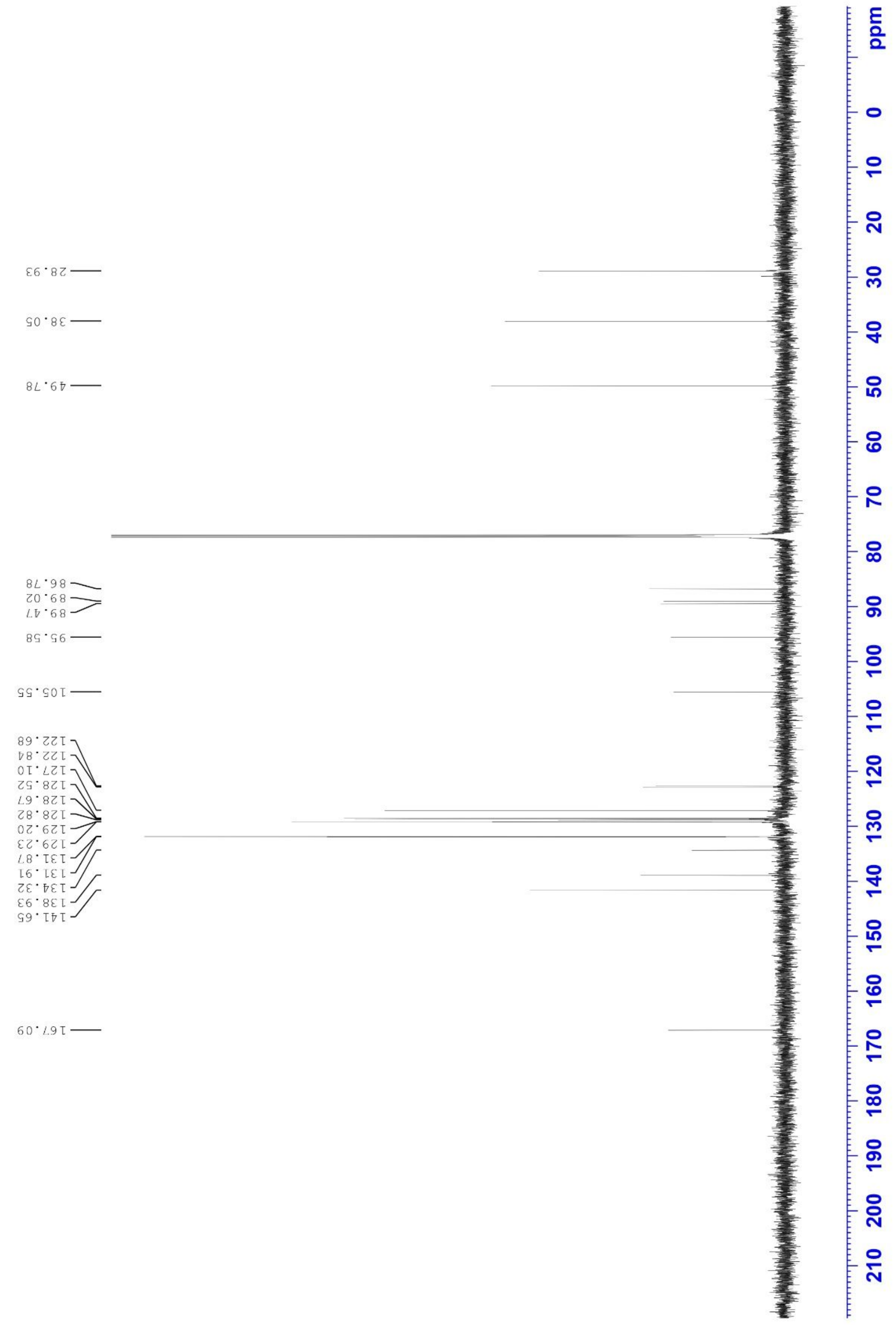

Figure S11. ^{13}C-NMR spectrum of RAR-BR. (150 MHz, $CDCl_3$).

Molecular Formula:	$C_{28}H_{22}N_4O$
Formula Weight:	430.50048
M+:	430.178813 Da
[M+H]+:	431.186638 Da

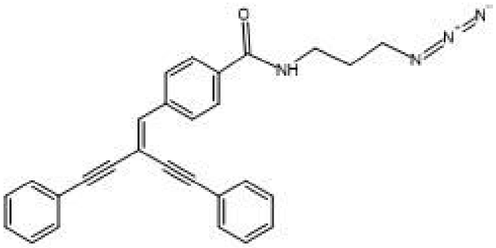

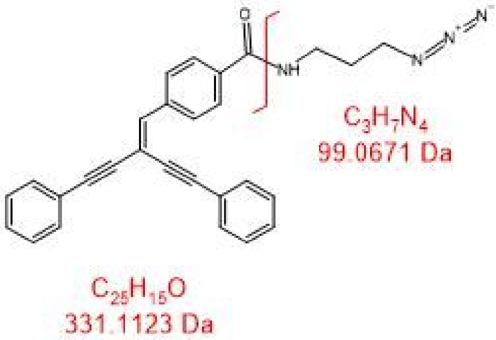

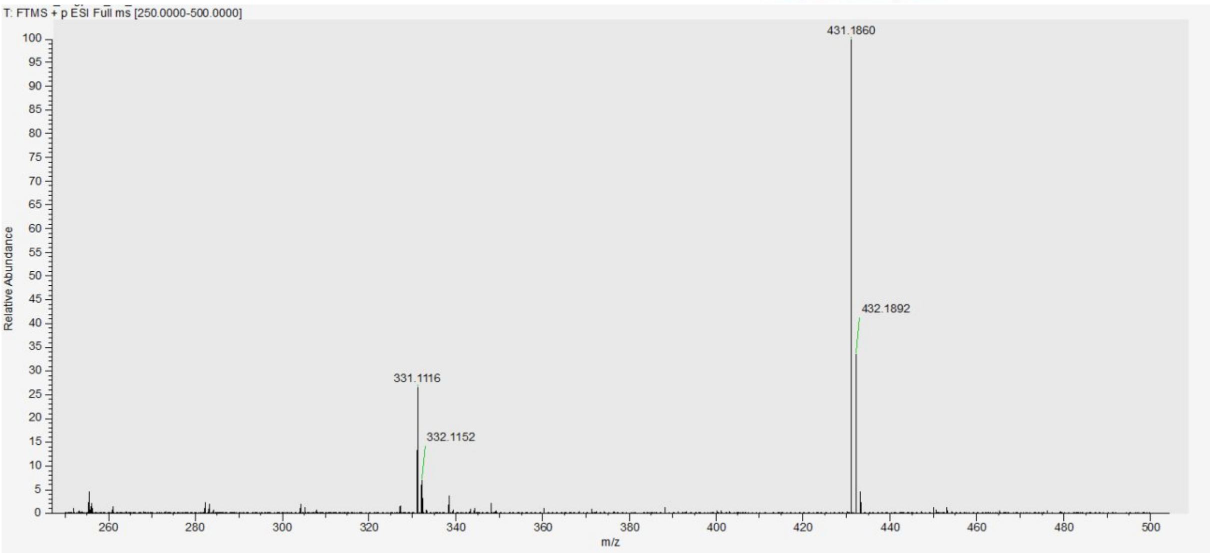

Figure S12. Mass spectrum of RAR-BR

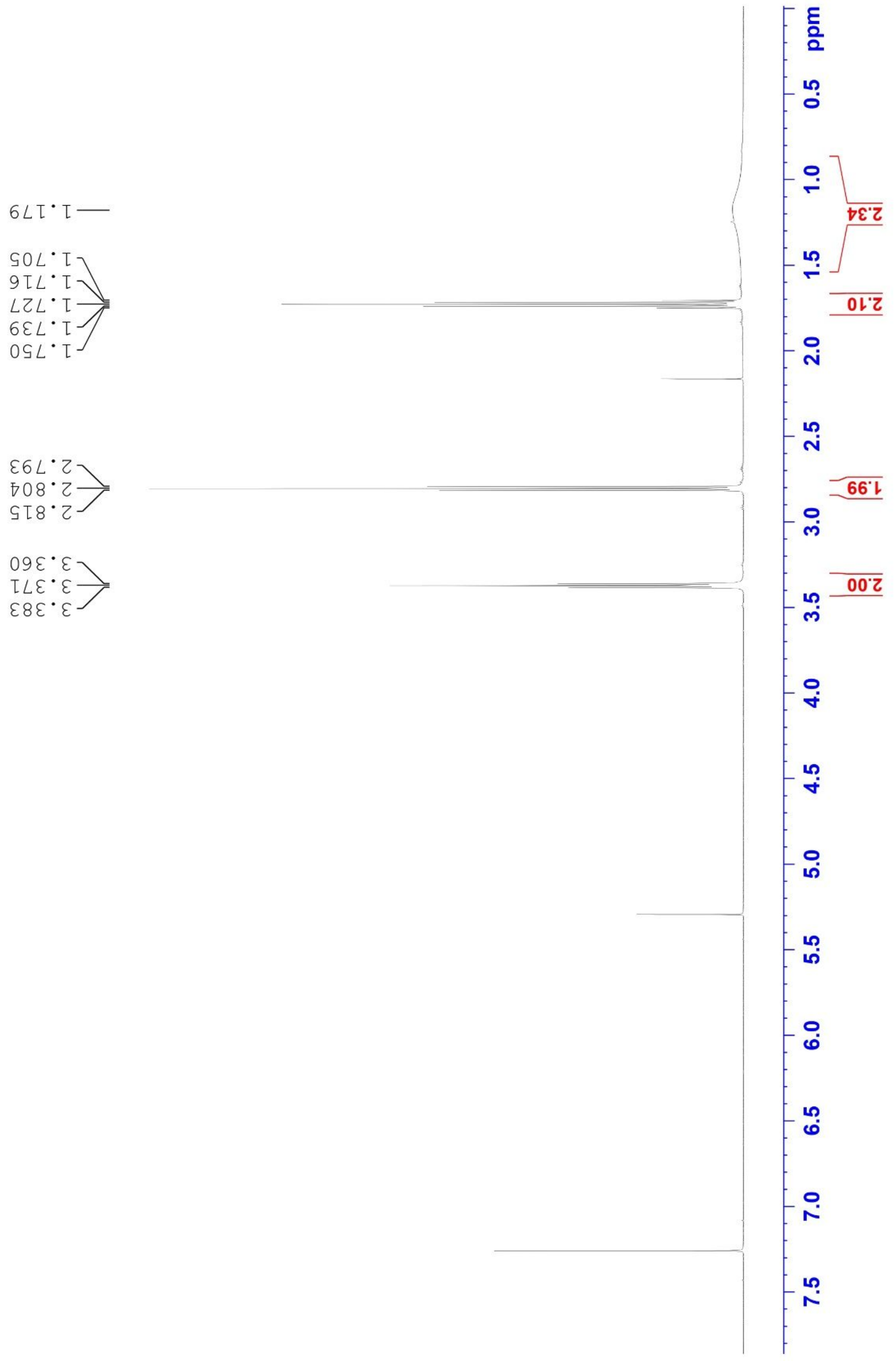

Figure S13. ^{1}H-NMR spectrum ^{1}H-NMR spectrum of 3-azidopropan-1-amine (7). (600 MHz, $CDCl_3$).

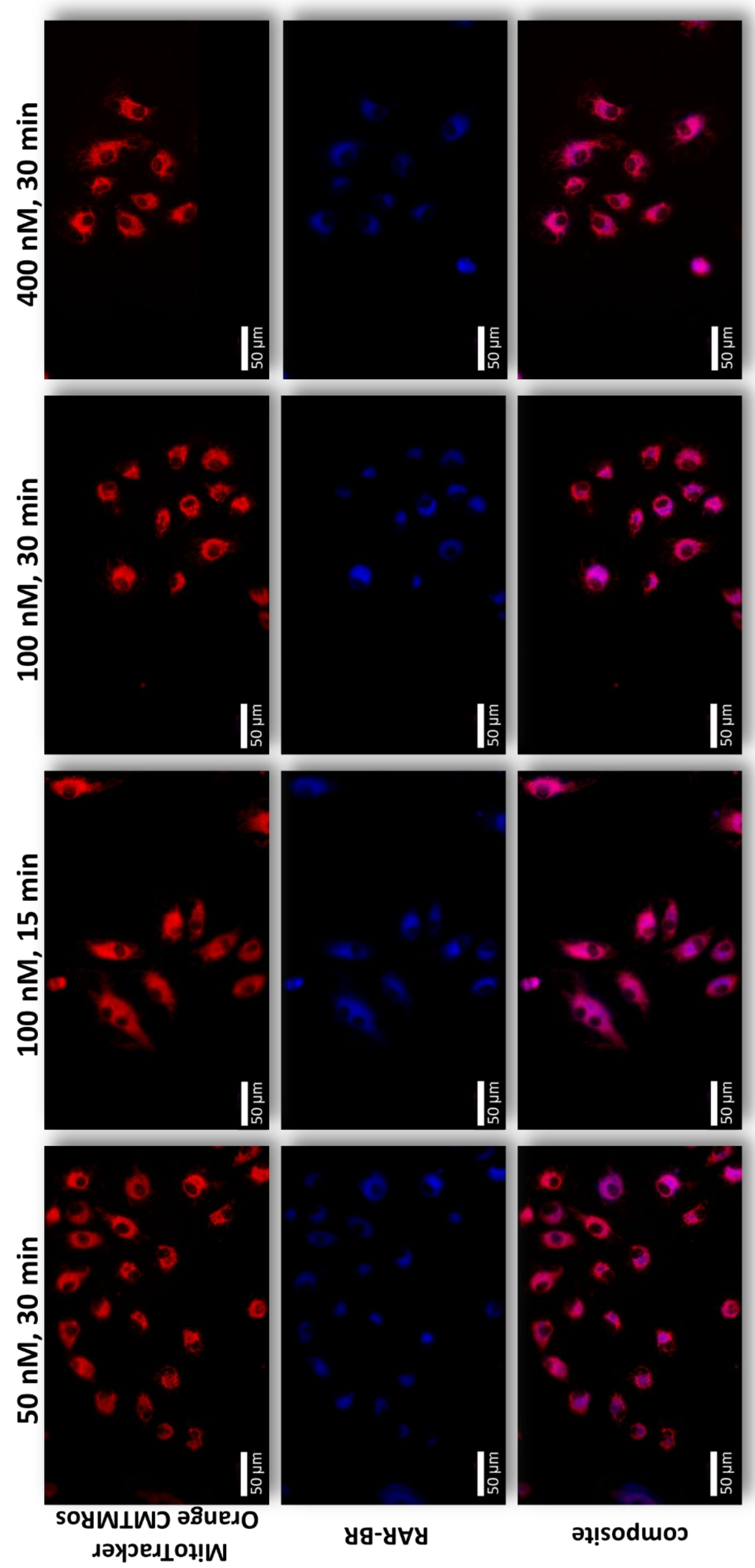

Figure S14. Fluorescence images of mitochondria in live HAEC cells stained with MitoTracker Orange CMTMRos and RAR-BR. Images were collected with a 40x objective

and Olympus Scan^R system. At the top the incubation time and concentration for both probes are given. Scale bar: 10 µm.

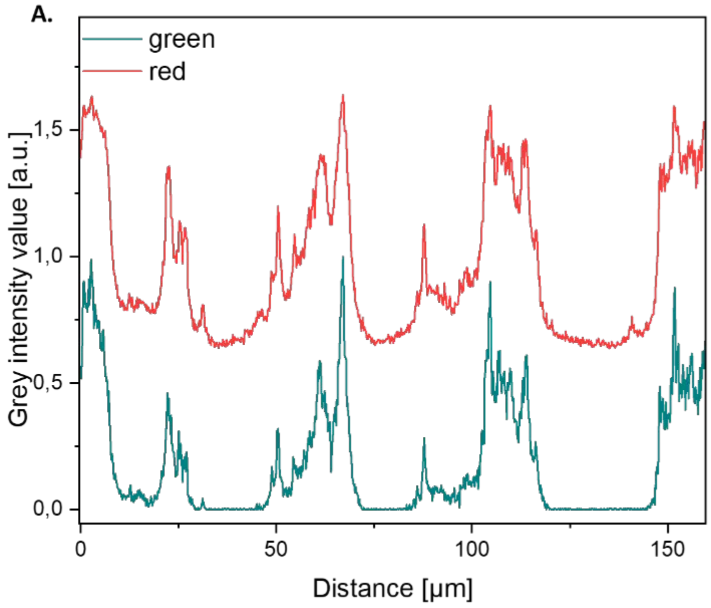

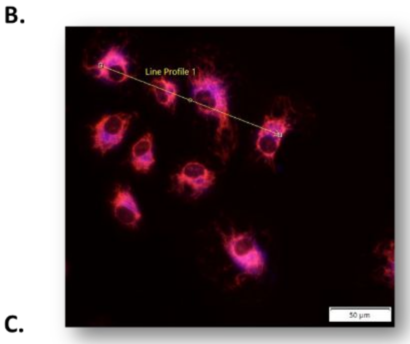

C.

Pearson Correlations	Value X [µm]	Green	Red
Value X [µm]	1	0,0611	0,0707*
Green	0,0611	1	0,96742*
Red	0,0707*	0,96742*	1

Figure S15. The fluorescence intensity line profile. A. The plot of fluorescence intensities along the intensity line profile (red: MitoTracker Orange CMTMRos, green: RAR-BR). B. Fluorescence images of mitochondria in live HAEC cells stained with MitoTracker Orange CMTMRos and RAR-BR with marked line profile. C. Pearson correlation coefficient table. * correlation is significant at 0.05.

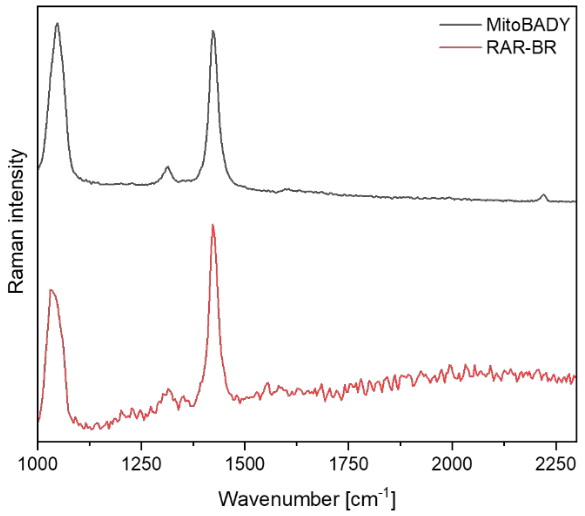

Figure S16. Raman spectra of MitoBADY and RAR-BR solution (1mM, DMSO).
